# Testing the Reproducibility of the Effects of Transcranial Direct Current Stimulation: Failure to Modulate Beauty Perception by Brain Stimulation

**DOI:** 10.3389/fnhum.2022.767344

**Published:** 2022-02-18

**Authors:** Kuri Takahashi, Yuko Yotsumoto

**Affiliations:** ^1^Department of Life Sciences, The University of Tokyo, Tokyo, Japan; ^2^Department of Psychology and Neurosciences, Leibniz Research Center for Working Environment and Human Factors, Dortmund, Germany; ^3^Department of Neuropsychology, Ruhr-Universität Bochum, Bochum, Germany

**Keywords:** tDCS, aesthetic judgment, reproducibility, prefrontal cortex, orbitofrontal cortex

## Abstract

Transcranial direct current stimulation (tDCS) has been recognized as a promising tool for investigating the causal relationship between specific brain areas of interest and behavior. However, the reproducibility of previous tDCS studies is often questioned because of failures in replication. This study focused on the effects of tDCS on one cognitive domain: beauty perception. To date, the modulation of beauty perception by tDCS has been shown in two studies: Cattaneo et al. (2014) and Nakamura and Kawabata (2015). Here, we aimed at replicating their studies and investigating the effects of tDCS on beauty perception using the following parameters: (1) cathodal stimulation over the medial prefrontal cortex (mPFC) (Nakamura and Kawabata, 2015); (2) anodal stimulation over the left dorsolateral prefrontal cortex (lDLPFC) (Cattaneo et al., 2014). We also performed a more focal stimulation targeting the orbitofrontal cortex (OFC) to determine the optimal stimulation site for modulating beauty perception (3). Participants rated the subjectively-perceived beauty of the images before and after the tDCS administration. We divided images into four clusters according to the obtained scores in our preliminary study and examined changes in beauty ratings in each image cluster separately to exclude factors, such as stimuli attributions that may reduce tDCS effects. The results showed no strong effects of tDCS with the same parameters as in previous studies on beauty rating scores in any image cluster. Likewise, anodal stimulation over the OFC did not result in a change in rating scores. In contrast to previous studies, the current study did not corroborate the effects of tDCS on beauty perception. Our findings provide evidence regarding the recent reproducibility issue of tDCS effects and suggest the possible inflation of its effects on cognitive domains.

## Introduction

Transcranial direct current stimulation (tDCS) is a major method that compensates for the drawbacks of neuroimaging techniques, i.e., the inability to prove causality between changes in brain activation and observed behaviors. By delivering a low-intensity, direct current to cortical areas, tDCS is considered to facilitate or inhibit spontaneous neuronal activity and modulate cortical area excitability localized below the stimulating electrode (Nitsche and Bolognini, 2013), inducing modulation of behavioral performance (Nitsche et al., 2008). Researchers have used tDCS to report causal evidence that a stimulated brain region is involved in the behavior of interest (Filmer et al., 2014). A sizeable number of studies have reported the effectiveness of prefrontal area stimulation in improving cognitive performance (Gladwin et al., 2012; Nelson et al., 2014; Ferrari et al., 2015; Abend et al., 2018). However, the replication of the effects of tDCS on some cognitive tasks, such as decision making, spatial attention, and probabilistic learning, has been failed in some studies (Koenigs et al., 2009; Seyed Majidi et al., 2017; Reteig et al., 2018). Publication bias (file drawer effect) may be one of the reasons for replication failures. For example, researchers may have selectively reported only significant results, although they conducted many preliminary studies with various electrode placements.

Here, we focused on the effects of tDCS on human beauty perception. The recently-emerging research field, called neuroaesthetics, has been trying to reveal the neural underpinnings of the process of making an aesthetic judgment by neuroscientific methods. Studies have reported the engagement of the prefrontal cortex, such as the dorsolateral prefrontal cortex (DLPFC) and the orbitofrontal cortex (OFC) in the aesthetic appreciation process. Specifically, the DLPFC was found to be activated when participants rated visual stimuli as beautiful (Cela-Conde et al., 2004), and the OFC showed significantly different changes in its activation, depending on whether participants rated stimuli beautiful or ugly (Kawabata and Zeki, 2004; Ishizu and Zeki, 2011, 2013). The DLPFC and the OFC were more active when participants rated stimuli in an aesthetic context compared to other contexts, such as brightness or pragmatism of stimuli, which may allow the presumption of functional specialization in aesthetic judgment (Cupchik et al., 2009; Ishizu and Zeki, 2013). Neuroaesthetics has also been using tDCS to test the relationship between these reported brain areas and beauty perception. To explore its causality, Cattaneo et al. (2014) applied anodal tDCS to the left dorsolateral prefrontal cortex (lDLPFC) and observed an increased level of aesthetic appreciation of representational images after stimulation (in the sample size of 12), assuming that the lDLPFC’s activity was more oriented toward judging stimuli in the aesthetic context by its activity enhancement. Nakamura and Kawabata (2015), on the other hand, applied the cathodal electrode on the mPFC, the anodal electrode on the left primary motor cortex (lPMC). They reported a significant decrease (in the sample size between 7–15 in one stimulation condition) only in participants’ beauty rating scores of visual stimuli, not in ugliness rating scores, possibly downregulating the mPFC’s activity in the perception of subjective experience of beauty.

However, there could be a potential issue which might affect the reproducibility of the previous studies. These outcomes did not result from a tDCS parameter that is optimized for modulation of beauty perception. Even if the targeted brain area is the same, different electrode montages can lead to different outcomes (Nasseri et al., 2015). The absence of tDCS montage specificity and the following heterogeneous results can also reduce the reproducibility of tDCS effects (Tremblay et al., 2014). One solution to this problem could be the assessment of the electrode montage’s efficacy at the neuronal level. For instance, some tDCS studies have paired up observed behavioral changes following stimulation and predicted current flow by a simulation software when reporting the results (Binney et al., 2018; Frings et al., 2018; Naka et al., 2018). In addition to deriving an electrode placement from previous studies, they referred to the estimated current flow distribution supporting their hypothesized tDCS effects. In Cattaneo et al. (2014)’s and Nakamura and Kawabata (2015)’s studies, however, no information was provided whether their tDCS montages were effective in stimulating the target brain areas. Therefore, the possibility cannot be excluded that there could be an optimal tDCS montage for stimulation of the mPFC and DLPFC.

Moreover, given the recent problems in reproducibility of tDCS effects, it is beneficial not only to support the existence of tDCS effects, but also to support its non-existence to prevent publication bias. One concern which could be related to the publication bias is the inflation of the effect sizes of earlier tDCS studies. This phenomenon was indeed shown empirically in the comparison between the average effect size of original studies and those of replications (Anderson et al., 2016; Trafimow et al., 2018; Open Science Collaboration, 2015). It indicates that applying sample size from original studies might lead to underpowered studies (Anderson et al., 2017).

In the present study, to test the reproducibility of the effects of tDCS on beauty perception and to find an optimal tDCS montage for modulation of beauty perception, we conceptually replicated Cattaneo et al. (2014) and Nakamura and Kawabata (2015) and took the following measures to design our study. First, before conducting the tDCS experiments, we performed a separate experiment to create a standardized image set. According to the obtained beauty rating, we divided images into four types of clusters: images with high, middle-high, middle-low, and low rating scores. The aim of creating clusters was to avoid that effects of tDCS are attenuated by the degree of perceived beauty of stimuli. The idea was based on the study which reported that modulation of emotion by tDCS was dependent on the amount of emotional experience evoked by affective stimuli (Abend et al., 2018).

Second, to locate an optimal stimulation site for the modulation of beauty perception, we stimulated three different areas: the mPFC from Nakamura and Kawabata (2015) in Experiment 2, the lDLPFC from Cattaneo et al. (2014) in Experiment 3, and the OFC in Experiment 4. At the same time, we assessed the efficacy of each tDCS montage by using the current flow simulation software. For Experiment 2 and 3, we simulated the current flow and strength in the target brain area in the MNI-defined standard brain template in case of using the electrode placement of previous studies (Soterix HD-Explore, Soterix Medical, NY, United States). For Experiment 4, we used an electrode montage which enables focal stimulation of the OFC with high current intensity in the MNI-defined standard brain template (Soterix HD-Targets, Soterix Medical, NY, United States). Correlational studies have suggested that the OFC’s activation was dependent on aesthetic judgment processes (Kawabata and Zeki, 2004; Ishizu and Zeki, 2011, 2013). Yet, the impact on OFC stimulation on beauty perception remains unexplored. In this simulation, the unknown current distribution and the electric field strength in the cortical ROI can be predicted in a three-dimensional head model using the well-established common tool, finite element method (FEM) (Datta et al., 2012). The accuracy of the modeling of current flow in the brain predicted by this FEM method is supported by measuring the induced current voltage and its flow in the head (Datta et al., 2013).

Third, we employed Bayes analysis to assess the behavioral changes after stimulation. The Bayes factor (BF) can indicate how strongly a hypothesis can be supported compared to the other (Kruschke, 2014). The Bayesian approach supports the null hypothesis (Rouder et al., 2009) and does not require the predetermination of a sample size, allowing us to obtain sufficient samples (Lee and Wagenmakers, 2014). Since the sample size can be determined independently from previous studies, the influence of the possible inflation of effect sizes can be minimized.

In summary, we aimed to explore an optimal tDCS montage for modulation of beauty perception by using the same parameters as Nakamura and Kawabata (2015) (cathodal tDCS of the mPFC, Experiment 2), Cattaneo et al. (2014) (anodal tDCS of the lDLPFC, Experiment 3) and applying tDCS over the OFC (Experiment 4). Furthermore, we conducted a Bayesian analysis to evaluate the degree to which tDCS effects were (or were not) supported.

## Experiment 1

### Materials and Methods

#### Participants

Participants included 31 healthy adults (15 males and 16 females, age in years: mean = 21.9, SD = 4.25). We recruited people until we had more participants than those in the preliminary studies of previous studies. All participants had normal or corrected-to-normal vision and provided written informed consent to participate in the experiment in accordance with the Declaration of Helsinki. They received monetary compensation for their participation. The institutional review boards of the University of Tokyo approved the protocol, and all experiments were carried out in accordance with the guidelines set by the Ethics Committee of the University of Tokyo.

#### Stimuli

The stimuli consisted of 185 images that were selected from the Open Affective Standardized Image Set (OASIS) (Kurdi et al., 2017). We chose images with valence rating of 3–7 on a scale from to 1–7 and arousal rating 3–5 on a scale from to 1–7. All images were photographs of landscapes, artifacts, and urban scenes. To avoid facial recognition brain mechanisms, images containing close views of humans were not included (Cattaneo et al., 2014). Stimuli were generated using MATLAB and the Psychophysics Toolbox (Brainard, 1997).

#### Procedure

**FIGURE 1:**
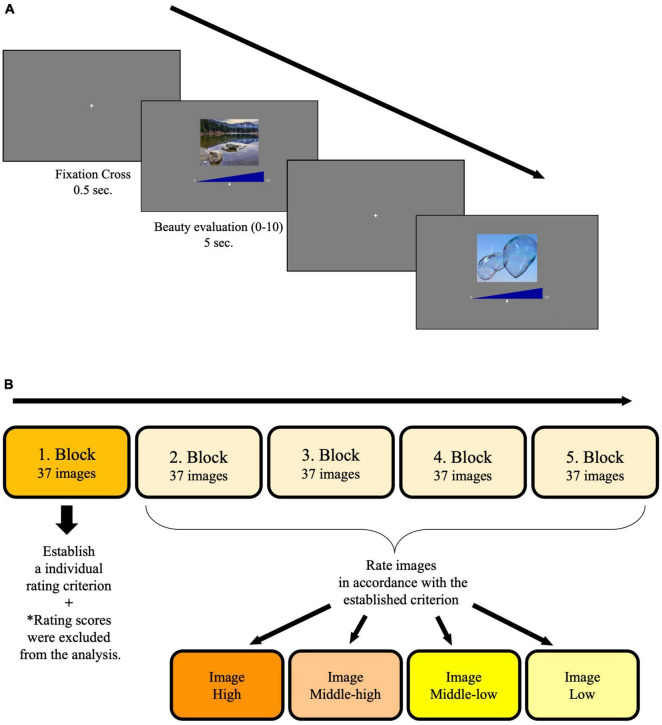
**(A)** Design of trials in the blocks. In each trial, participants had to rate the level of beauty they perceived from an image by moving a small triangle along the scale. The left end of the scale corresponded to 0 (not beautiful at all), and the right end of the scale corresponded to 10 (absolutely beautiful). **(B)** Experimental design. Participants were told to establish an individual rating criterion in the first block. Images in the following blocks were rated in accordance with the established criterion and divided into four clusters.

Images were presented on a gamma-corrected 21.5″ iMac display (4,096 pixels × 2,304 pixels) and were viewed from a distance of approximately 57.3 cm in the normally-lit and silent testing room. Each trial started with a fixation cross presented for 0.5 s on a gray background (8.65 cd/m^2^) after an image and a blue rating scale appeared. Figure 1A shows the trial design. Participants were informed that the scale was supposed to measure how much beauty they perceived from the presented image, the left end of the scale corresponded to the minimum feeling of beauty (0), and the right end of the scale corresponded to the maximum feeling of beauty (10). Participants were instructed to move the small triangle along the scale by pressing the right or left arrow key on the keyboard and to confirm their rating by pressing the upper arrow key. Using the entire range of the scale was encouraged. Each image subtended a visual angle of 8.5° × 6.8°, was presented for 5 s, and they had to confirm their rating within the image presentation duration. The experiment consisted of 5 blocks, each of which contained 37 images (Figure 1B). At the beginning of the experiment, participants were encouraged to establish a rating criterion within the first block and rate images of the following blocks in accordance with that criterion. Participants were told that the rating criterion could be subjective. The order of the image presentation was randomized across the participants. The entire experiment took approximately 20 min.

### Results

**FIGURE 2:**
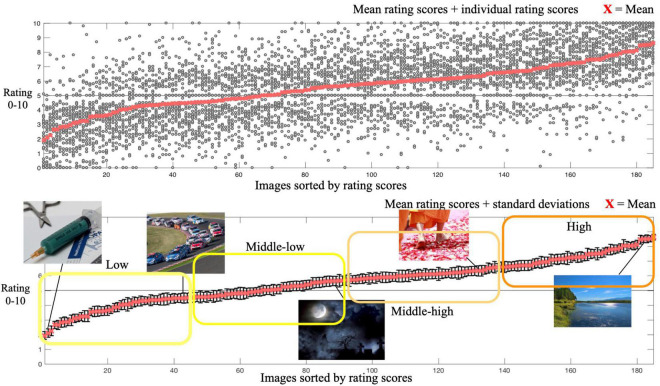
Red crosses in both panels represent mean rating scores of images in Experiment 1. Individual rating scores are plotted in the upper panel. Representative rated images at each data point are shown in the bottom panel. Error bars represent standard deviations (SD) of rating scores.

Considering the stability of rating scores, we excluded the rating scores of images presented in the first block or those which were not rated within 5 s. Figure 2 shows the ordered mean rating scores of the images. After excluding a duplicate image (Image 169), we divided 184 images (Image 169 was excluded) into 4 clusters based on the acquired mean rating scores: high (mean rating score = 7.4, SD = 0.6), middle-high (mean rating score = 6.1, SD = 0.24), middle-low (mean rating score = 5.09, SD = 0.38), and low (mean rating score = 3.73, SD = 0.73). Each cluster contained 46 images.

## Experiment 2

In this experiment, we replicated Nakamura and Kawabata (2015) by applying cathodal tDCS over the mPFC.

### Materials and Methods

#### Participants

Participants included 33 healthy adults aged 18–27 years (19 males and 14 females, age in years: mean = 19.85, SD = 2.15). None of them participated in Experiment 1. The data collection was not stopped until the number of participants was almost twice as large as that in previous studies. All participants had normal or corrected-to-normal vision and provided written informed consent to participate in the experiment in accordance with the Declaration of Helsinki. They received monetary compensation for their participation. The institutional review boards of the University of Tokyo approved the protocol, and all experiments were carried out in accordance with the guidelines set by the Ethics Committee of the University of Tokyo. Four participants were excluded from the analysis due to low adherence to the task, incomplete participation in the experiment, and high impedance level.

#### Transcranial Direct Current Stimulation

**FIGURE 3:**
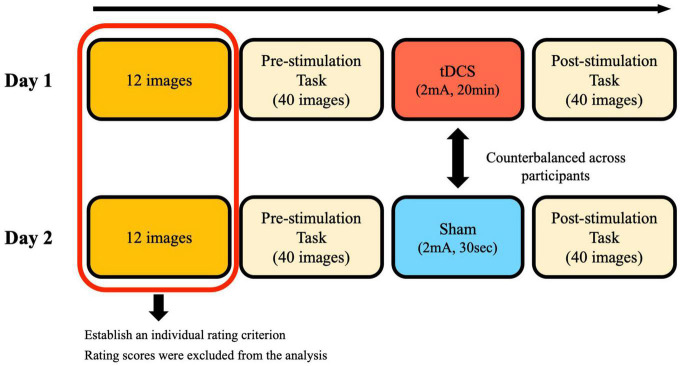
Experimental design of Experiments 2, 3, and 4. By rating 12 images, participants established an individual rating criterion. They were told to rate 40 images in the pre-stimulation task and the post-stimulation task in accordance with the established criterion.

A battery-driven, constant current stimulator (Eldith, Neuroconn, Ilmenau, Germany) delivered the tDCS through rubber electrodes placed in saline-soaked sponges. The size of the electrodes was 5 cm × 7 cm. The cathodal electrode was placed over the mPFC localized as the middle point between Fp1 and Fp2 and the glabella in the standard 10–20 electroencephalography (EEG) system. The anodal electrode was placed over the lPMC localized as C3 (Nakamura and Kawabata, 2015). Figure 3 shows the experimental design (the experimental design was the same as in Experiments 3 and 4). Each participant underwent two stimulation sessions: a real stimulation condition (tDCS condition) and a sham stimulation condition (sham condition). To avoid a carry-over effect, sessions were separated by at least 2 days (Nitsche et al., 2008). The order of the sessions was counterbalanced across participants. In the tDCS condition, the stimulation intensity was set at 2 mA, and the stimulation lasted 20 min. In the sham condition, the stimulation intensity was the same as in the tDCS stimulation condition, but the stimulator was on only for 30 s, so that participants felt the initial itching sensation and assumed that they would be stimulated for the same duration in both conditions. This was a single-blind study.

#### Stimuli

A total of 184 OASIS images were divided into 4 clusters according to the rating scores from Experiment 1 (high, middle-high, middle-low, and low) were used as stimuli. Each cluster contained 46 images. Stimuli were generated using the same apparatus as in Experiment 1.

#### Procedure

Participants were first instructed to establish their own rating criteria by evaluating 12 images that were also considered as a practice. Participants were told that the criterion should be subjective. These 12 images consisted of 3 randomly-chosen images from each of the 4 clusters. They were always newly-generated at the beginning of the session and were not used in the real session. Participants then rated 40 images according to their own criteria (pre-stimulation rating task). A total of 10 images were randomly-chosen from each of the 4 clusters to create a set of 40 images. After the pre-stimulation rating task, the mPFC and the lPMC were localized, and electrodes were placed over the participants’ heads. Before the stimulation started, participants were told to be seated in a relaxed position and not to fall asleep during stimulation. Immediately after stimulation ended, participants started rating another new set of 40 images (10 images from each of the 4 clusters) (post-stimulation task). All images were presented in a random sequence across participants, and each image was presented only once. The entire experiment took approximately 1 h.

#### Data Analysis

The data were analyzed using MATLAB programming language and JASP (JASP Team, 2021). Rating scores of the first 12 images of both tDCS and sham conditions were excluded from the analysis because they were rated before establishing a rating criterion. To evaluate the effect of tDCS on beauty perception, we subtracted pre-stimulation rating scores from those of post-stimulation for each participant. Subsequently, we compared the mean of these rating score differences of all participants between the tDCS and sham conditions in each image cluster. Bayesian analogs of within-subjects comparisons of the mean were conducted using JASP with the independent Cauchy distribution (location parameter = 0, scale parameter = 0.707) as the prior (Lee and Wagenmakers, 2014); therefore, we computed Bayes Factor in terms of evidence for the alternative hypothesis as well as the null hypothesis. To classify the strength of the evidence, we employed the scheme of Lee and Wagenmakers (2014). Additionally, we performed the null hypothesis significant testing (NHST) for every Bayesian analysis for comparisons with previous studies (Table 1).

**TABLE 1:** Difference of mean rating scores between pre- and post-stimulation in the high, middle-high, middle-low, and low image cluster in the tDCS and sham condition.

		tDCS (post-pre)		Sham (post-pre)		*t*	*p*	Cohen’s *d*
		*M*	SD	*M*	SD	*t*(28)		
Experiment 2	High	–0.11	0.907	–0.08	0.895	–0.14	0.89	–0.034
	Middle-high	–0.197	0.853	0.1223	0.757	–1.412	0.169	–0.396
	Middle-low	–0.053	0.936	0.14	0.933	–0.92	0.365	–0.207
	Low	–0.007	0.997	0.111	0.732	–0.559	0.58	–0.135
						*t*(28)		
Experiment 3	High	–0.098	0.584	–0.214	0.82	0.606	0.549	0.163
	Middle-high	–0.066	0.856	–0.127	0.749	0.264	0.794	0.076
	Middle-low	0.146	0.813	0.044	0.988	0.522	0.606	0.113
	Low	0.159	0.879	0.277	1.005	–0.533	0.598	–0.125
						*t*(29)		
Experiment 4	High	–0.186	0.685	–0.411	0.64	1.323	0.196	0.34
	Middle-high	0.095	0.813	–0.252	0.819	1.703	0.099	0.426
	Middle-low	–0.102	1.044	0.178	1	–1.098	0.281	–0.274
	Low	0.029	0.666	–0.249	0.784	1.763	0.088	0.382

### Results

**FIGURE 4:**
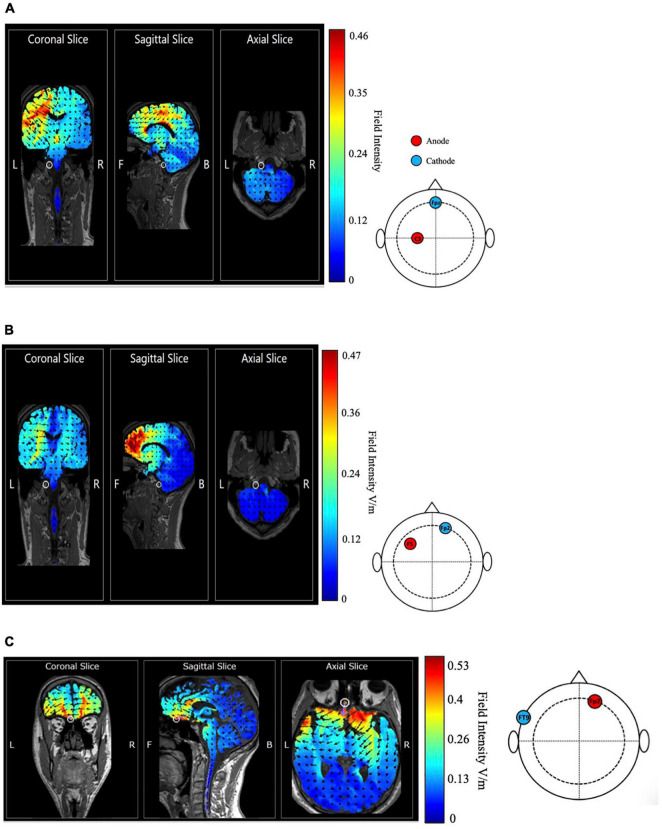
Simulated distribution of the tDCS induced current in the brain with the montage of **(A)** Nakamura and Kawabata (2015), **(B)** Cattaneo et al. (2014) and **(C)** which was designed to maximally stimulate the OFC in the coronal, sagittal, and axial view. Red area indicates a high current field intensity, and blue area indicates a low current field intensity. Arrows show predicted current flow.

**FIGURE 5:**
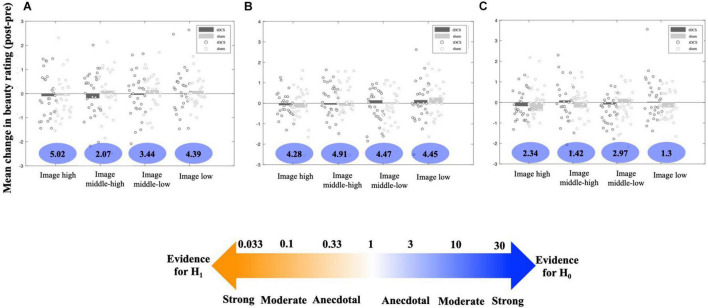
Mean difference in rating scores between post- and pre-stimulation task in tDCS and sham conditions of each image cluster in **(A)** Experiment 2, **(B)** Experiment 3 and **(C)** Experiment 4. Bars represent the mean difference in rating scores of all participants and dots represent the mean rating score differences for each participant. Numbers below the bars are BFs.

Figure 4A depicts the simulated current flow and intensity as modeled using HD-Explore software (Soterix Medical, NY, United States). Since the software can simulate only the current flow from a location of the 10–20 system, we set the place of cathodal electrode (middle point between Fp1, Fp2, and the glabella) as Fpz and simulated the expected current flow and voltage with the head template. It shows that the induced current appears to spread over to the broad range. The possibility of relatively less focal stimulation cannot be ruled out, indicating that the other brain areas might have been affected by the stimulation as well and tDCS effects over the mPFC could have been attenuated. Contrary to the finding of Nakamura and Kawabata (2015), we found no strong effects of cathodal tDCS over the mPFC on beauty perception compared to the sham stimulation condition in any clusters (Figure 5A). All BFs indicated that the obtained data would fit better under the null hypothesis than under the alternative hypothesis. The null hypothesis was supported more than the alternative one.

## Experiment 3

In this experiment, we replicated Cattaneo et al. (2014) by applying anodal tDCS over the lDLPFC.

### Materials and Methods

#### Participants

Participants included 30 healthy adults aged 18–21 years (18 males and 12 females, age in years: mean = 19.2, SD = 0.85). None of them participated in Experiments 1 or 2. The data collection was not stopped until the number of participants was almost twice as large as that in previous studies. All participants had normal or corrected-to-normal vision and provided written informed consent to participate in the experiment in accordance with the Declaration of Helsinki. They received monetary compensation for their participation. The institutional review boards of the University of Tokyo approved the protocol, and all experiments were carried out in accordance with the guidelines set by the Ethics Committee of the University of Tokyo. One participant was excluded from the analysis because of the high impedance level.

#### Transcranial Direct Current Stimulation

The anodal electrode was placed over the 1DLPFC localized as the middle point between F3 and F5 in the standard 10–20 EEG system, and the cathodal electrode was placed over the right supraorbital region (Cattaneo et al., 2014). The stimulation intensity, duration for both tDCS and sham conditions, and the apparatus were the same as in Experiment 2.

### Results

Figure 4B is the simulated current flow and intensity as modeled using HD-Explore software (Soterix Medical, NY, United States). Since the software can simulate only the current flow from a location of 10–20 system, we set the place of anodal electrode (middle point between F3 and F5) as F5. It shows that the high field intensity (max. 0.48 V/m) concentrates on the frontal lobe, indicating the montage of Cattaneo et al. (2014) could be able to induce relatively focal stimulation of the target area.

While Cattaneo et al. (2014) reported a significant change in beauty perception after stimulation, no evidence of change in rating scores of any image clusters was found in our study (Figure 5B). In all image clusters, non-effect of tDCS on beauty perception was moderately supported.

## Experiment 4

In this experiment, we applied anodal tDCS over the OFC to explore its causal involvement in beauty perception.

### Materials and Methods

#### Participants

Participants included 32 healthy adults aged 18–31 years (17 males and 15 females, age in years: mean = 19.81, SD = 2.31). None of them participated in Experiments 1, 2, or 3. The data collection was not stopped until the number of participants was almost twice as large as that in previous studies. All participants had normal or corrected-to-normal vision and provided written informed consent to participate in the experiment in accordance with the Declaration of Helsinki. They received monetary compensation for their participation. The institutional review boards of the University of Tokyo approved the protocol, and all experiments were carried out in accordance with the guidelines set by the Ethics Committee of the University of Tokyo. Two participants were excluded from the analysis because of incomplete participation in the experiment.

#### Transcranial Direct Current Stimulation

For the OFC stimulation, we determined the electrode montage using HD-Targets (Soterix Medical, NY, United States), which suggests optimal electrode placement for the desired target area. According to the simulation results, the anodal electrode was placed over Fp2, and the cathodal electrode was placed over FT9 in the standard 10–20 EEG system. The stimulation intensity, duration for both tDCS and sham conditions, and the apparatus were the same as in Experiment 2.

### Results

Figure 4C shows that the maximal field intensity (0.53 V/m) is located in the OFC marked by a white circle. Among the all three tDCS montages we have tested, the montage for stimulation of the OFC showed the highest field intensity, suggesting the relative focal stimulation on the target area could have been achieved.

However, again, we found no evidence supporting a change in rating scores after tDCS (Figure 5C). This finding was consistent across all image clusters (BF > 1).

## Discussion

In this series of studies, we evaluated the effects of tDCS on beauty perception using three different electrode montage on beauty perception: (1) cathodal tDCS over the mPFC (Nakamura and Kawabata, 2015), (2) anodal tDCS over the lDLPFC (Cattaneo et al., 2014), and (3) anodal tDCS over the OFC. Our study idea was derived from Cattaneo et al. (2014) and Nakamura and Kawabata (2015). We administered stimulation with the same intensity (2 mA) as that of their studies and tested whether tDCS led to a change in beauty rating scores, compared to the sham condition.

Our findings contradict previous studies. Independent of the image cluster type, no solid evidence for the effect of cathodal tDCS over the mPFC and anodal tDCS over the lDLPFC on participants’ perception of beauty was obtained. Some factors may contribute to these conflicting results. First, our study was not replicated exactly. To qualitatively evaluate the effectiveness of electrode placements in their studies, we used a different image set. Second, some studies have suggested that individual differences in behavioral modulation appear to be associated with the magnitude of change in brain activity induced by tDCS. For instance, Falcone et al. (2018) demonstrated that the enhancement of visual attentional task performance, which was not evident at the group level, was correlated with individual differential neuronal activation. This result was also supported by the finding that the degree of behavioral modulation was significantly correlated with the current density in the targeted area (Kim et al., 2014). Factors, such as individual differences in head size and strength of functional connectivity between brain regions are also considered to lead to heterogeneous outcomes of tDCS (Krause and Kadosh, 2014). Therefore, it may be reasonable to speculate that the effects of tDCS on behavior were not observed in our behavioral analysis due to these individual differences in the target area’s activation. As for the tDCS effects over the OFC, we found no effects of tDCS on the beauty ratings in any image clusters. Considering the failure in replication of tDCS effects over the mPFC in our study, it seems consistent that neither tDCS over the mPFC nor over the OFC resulted in modulation of beauty perception.

Turning the focus to the simulation results of induced current flow, adhering to the intention of Nakamura and Kawabata (2015), the cathodal stimulation over the mPFC led to a low focality in stimulation of the targeted area. The current flowing from the lPMC appeared to spread over a broader area compared to the other stimulation conditions and might have affected the other brain areas as well. It suggests the possibility that the modulation of beauty perception observed in the previous study may not be explained solely by effects of cathodal tDCS over the mPFC. This low focality could have diluted the effects of tDCS and resulted in the low reproducibility of the previous study.

However, the simulation results may also highlight the difficulty in replicating the effects of tDCS on the prefrontal area. The induced maximal intensity was the highest in the OFC stimulation condition, according to the current flow simulation. Nevertheless, tDCS over the OFC did not affect participants’ perception of beauty in our study. Additionally, stimulation over the lDLPFC did not change beauty ratings despite the relatively focal stimulated area. The results may indicate the difficulty in hypothesizing the tDCS effects by the predicted stimulation intensity pattern alone. Nonetheless, assessing the predicted current flow pattern along with tDCS effects on behavior is expected to provide a new perspective to the conventional way of discussing tDCS effects, which often has been lacking—a neuronal level perspective. Recent studies using tDCS have shown that behavioral changes following stimulation and simulated current flow are paired up when reporting the results (Binney et al., 2018; Frings et al., 2018; Naka et al., 2018). In addition to deriving an electrode placement from previous studies, these referred to the estimated current flow distribution as evidence for their hypothesized tDCS effects. In addition to measuring current flow by brain imaging, simulation software should contribute to enhancing the reproducibility of tDCS effects in future studies.

There is another possibility that also needs to be discussed here, namely the involvement of other brain areas in beauty perception in the present study. For instance, Silveira et al. (2015) indicated that aesthetically preferred paintings elicited activation of the bilateral anterior cingulate cortex (ACC) and the right precuneus and the bilateral TPJ, which are not the targets of the stimulation in the current study. Moreover, Chuan-Peng et al. (2020) investigated the existence of a common neural basis for beauty perception by using different forms of stimuli (artworks and faces). However, they failed to find a common brain area, while the anterior medial prefrontal cortex (aMPFC) was activated for the beauty of artworks, and the left ventral striatum was activated for the beauty of face stimuli. These findings may indicate the complexity of defining beauty. In the present study, we instructed participants to rate stimuli based on a fixed criterion that they predetermined. However, brain areas involved in the rating task may have been different across participants depending on what kind of definition of beauty they applied, and the consistent stimulation place across participants may not have been effective in modulating beauty perception.

One of the limitations of our study was that the applied current density in Experiment 2 was lower than that in the study by Nakamura and Kawabata (2015). While they applied tDCS with 5 cm × 5 cm electrodes (0.08 mA/cm^2^), we applied tDCS with 5 cm × 7 cm electrodes (0.057 mA/cm^2^). Indeed, it has been shown that higher current density/intensity induces greater changes in cortical excitability (Nitsche and Paulus, 2000). A meta-analysis also indicated the significant effect of current density on performance in cognitive tasks (Dedoncker et al., 2016). Our replication failure of Nakamura and Kawabata (2015)’s study, therefore, could have resulted from the insufficient amount of induced current in the target area. However, the effect of the applied current density remains inconclusive. Bastani and Jaberzadeh (2013) and Ho et al. (2016) found no linear relationship between the current density/intensity and cortical excitability, showing that a higher current density does not necessarily induce a greater increase in cortical excitability compared to lower current density. Nonetheless, it should be noted that the different amounts of induced current density could explain the non-replicated results of the present study.

Another limitation was that only the effects of anodal stimulation of the OFC were examined which might have resulted in the null results in our study. In Nakamura and Kawabata’s study, only cathodal stimulation of the mPFC was found to modulate the perception of beautiful stimuli. The effects of cathodal stimulation of the OFC are needed to be examined in future studies.

## Conclusion

In summary, the results presented here did not replicate the studies by Cattaneo et al. (2014) and Nakamura and Kawabata (2015). Additionally, focal anodal stimulation over the OFC did not exert its effect. Therefore, the effects of tDCS on human beauty perception remain unclear. However, our findings do not reject the utility of tDCS in investigating neuronal mechanism of cognition. Instead, we aimed to highlight the possibility that tDCS effects on some cognitive domains could be inflated due to noise in data or interpretations of outcome. Future research should determine the sample size based on power analysis in case of using the frequentist approach for data analysis, select stimulation location corroborated by data at the neuronal level, and most importantly, report all results, including null.

We confirm that we have reported all measures, conditions, data exclusions, and how we determined our sample sizes for all experiments.

## Data Availability Statement

The datasets presented in this study can be found in online repositories. The names of the repository/repositories and accession number(s) can be found below: OSF database (DOI 10.17605/OSF.IO/8QWY3).

## Ethics Statement

The studies involving human participants were reviewed and approved by Ethics Committee of the University of Tokyo. The patients/participants provided their written informed consent to participate in this study.

## Author Contributions

KT and YY conceived and designed the experiments and wrote the manuscript. KT performed the experiments and analyzed the data. Both authors contributed to the article and approved the submitted version.

## Conflict of Interest

The authors declare that the research was conducted in the absence of any commercial or financial relationships that could be construed as a potential conflict of interest.

## Publisher’s Note

All claims expressed in this article are solely those of the authors and do not necessarily represent those of their affiliated organizations, or those of the publisher, the editors and the reviewers. Any product that may be evaluated in this article, or claim that may be made by its manufacturer, is not guaranteed or endorsed by the publisher.
